# Supplementary material for: Are carbon emissions trading and green financial instruments synergistic? -Comprehensive quantitative research based on content analysis
Source: PLoS One. 2024 Mar 7;19(3):e0298601. doi: 10.1371/journal.pone.0298601 (PMC10919601; doi:10.1371/journal.pone.0298601)
Supplement: S1 Data — (ZIP) [file pone.0298601.s001.zip › Data/地级市Z_F工作报告.zip!/░▓╗╒/╗┤▒▒╩╨/└┤╫╘╣1⁄2╓┌║┼í╛┬φ┐╦╩2╛▌═°í┐_2008.docx]

各位代表:

     我代表市人民政府,向大会报告工作,请予审议,并请市政协委员和其他列席人员提出意见。

     ****一、真抓实干,全市经济社会发展取得新成绩****

    十三届人民政府在市委的坚强领导下,在市人大、市政协的监督支持下,团结和带领全市人民高举邓小平理论和“三个代表”重要思想伟大旗帜,认真贯彻党的十六大及省、市党代会精神,深入落实科学发展观,团结拼搏,创新实干,扎实推进改革开放和现代化建设,迈上了全面建设小康社会的新征程。

     五年来,我们科学定位,围绕建设现代化工业城市,大力实施工业强市、开放带动、创新推动、全民创业、城乡一体化和可持续发展战略。审时度势,作出了经济发展处于调整期、各项改革处于过渡期、结构调整处于转型期、全民创业和县域经济发展处于培育期的科学判断,扬长补短,攻坚克难,着力做好打基础、管长远、增后劲的工作。致力和谐,始终坚持执政为民,加强社会建设,实施民生工程,维护社会稳定,经济社会发展取得了明显成效。

     国民经济持续增长。全市地区生产总值和财政收入年均分别增长12%和19.7%。工业经济主导地位更加突出。制定并实施“3588”工业振兴计划,抓企业、抓投资,加强技术创新和管理创新,实施名牌战略和质量兴企,大力培育市场主体和优势产业,不仅发展速度、质量、效益同步增长,而且实现了发展后劲的增强和国家名牌产品零的突破。2007年,规模以上工业企业262家,比2002年增加161家;实现增加值117亿元,年均增长14.1%,占GDP的比重比2002年提高5.5个百分点;实现利税31亿元,是2002年的2.4倍。五年累计完成工业投资305.7亿元,年均增长46.9%;共有中国名牌产品1个、驰名商标1件和7个国家免检产品。农业农村经济稳步发展。组织实施小麦高产攻关活动,粮食单产、总产连创历史新高。实施畜牧富民工程,被列为全省生猪养殖核心区、奶牛生产示范区。大力发展果蔬等高效经济作物,“菜篮子”工程连续16年全省第一。加强绿色长廊建设和荒山绿化等,林业建设取得新成绩。加速推进农业产业化经营,龙头企业达120家,其中8家被评为省级龙头企业。积极发展劳务经济,农村劳务输出规模达35.5万人,农民工资性收入占纯收入比重由2002年的52.5%提高到65%。第三产业稳步提高。适应消费升级需求,加强市场建设,发展各类批发市场190个,商业网点达到1.7万个;城乡市场繁荣活跃,社会消费品零售总额年均增长12.9%。县域经济增势强劲。理顺利益关系,倾斜项目布局,促进县区产业升级和实力提升。2007年县区地区生产总值平均增长15.5%,对全市经济增长贡献率达52.3%。

    改革开放步伐加快。推进农村综合改革,全面取消农业税,完成区划调整,并初步建立基层管理新体制和工作运行新机制。推进国企改革,市属国有企业改革基本完成,累计支付改制成本8.9亿元,2.4万名职工通过及时调整劳动关系在新企业重新就业;现代企业制度加快建立,雷鸣科化、恒源煤电、飞亚纺织成功上市。推进财政体制改革,调整理顺市、县(区)财政体制,初步建立公共财政管理新框架。推进公用事业改革,中北巴士、华润燃气运行良好。开展全民创业行动,组织实施中小企业成长工程,加强银企对接,成功举办两届专利技术推介会,非公有制经济加快发展,5年新增民营企业3409家,个体工商户2.6万户。

     注重理性务实招商,不断加大产业、驻点和节会招商力度,成功举办两届煤矿机械投资贸易洽谈会及其他一系列招商活动,山东六和、天津宝迪、台湾旺味、浙江维科、深圳华孚等一批大企业相继落户淮北,五年累计引进市外资金265.1亿元人民币,外资1.96亿美元,年均分别增长58.2%和24.8%。举全市之力加快开发区和工业园区建设,“三区两园”格局已经形成,其中龙湖工业项目区16平方公里基础设施建设基本完成,重点产业加速集聚;县区开发区和工业园区建设加快,承载力和发展力进一步增强。对外贸易平稳增长,五年累计完成进出口总额2.8亿美元,其中出口2.4亿美元。外事、侨务等工作全面加强,与韩国抱川等国际友城交流进一步深入。

     发展后劲显著增强。以省“861”行动计划和市重点工程建设为统领,强化项目带动,优化投资结构,积蓄了加快发展的能量和后劲。五年累计完成城镇固定资产投资441亿元,年均增长38%。强力推进工业项目建设,新的经济增长点加速形成。煤化工产业强势成长,临涣焦化一期前3号焦炉投产,4号焦炉加快建设,焦化二期正在环评。煤电基地建设加快,涡北等新建矿井、许疃等扩建矿井以及500千伏变电站等项目竣工投产;卧龙湖、孙疃等矿井和临涣选煤厂等新建、扩建项目进展顺利。非煤产业加速壮大,东磁电子8条化成箔生产线、富马酸等项目建成投产,华润雪花啤酒扩建、龙波电器二期等项目建成,杜集开发区矿山机械装备制造业加速集聚。强力破解要素瓶颈制约,重大问题解决取得突破性进展。合徐、连霍高速公路连接线建成,濉永路竣工通车,省道202线启动改扩建,五年累计新建、改建干线公路7条,总里程100公里。实施建设用地置换,统筹分配使用指标,从严控制投资强度,开展工业用地“招拍挂”,集约节约用地水平不断提高。启动节水型社会和节水型城市试点建设,开工兴建“淮水北调”管道输水工程。发挥电力优势,城乡用电同网同价,大用户直购试点工作有序推进。开展新、老“五小”企业和重点污染源整治,资源环境质量改善。重组市国有资产运营公司,组建市信用担保公司,做大做强城投公司,加强政银企对接,要素支撑发展的能力不断增强。

     城乡面貌变化显著。大力推进城市建设。《淮北市城市总体规划(2006-2020年)》通过了国务院审批。实施城市东进、西延、南扩,建成区面积达到68平方公里,规模比2002年扩大1倍以上。努力改善居民居住条件,完成投资51.4亿元,建房397.6万平方米;加强市政基础设施建设,新建和改造淮海路、濉溪路等30多条道路,老濉河、相阳沟等13条骨干河沟,以及污水管网、污水处理厂,开工建设天然气利用等项目,城市功能不断增强。深入开展文明城市和园林城市创建活动,精神文明建设和城市管理始终走在全省前列,荣获“国家园林城市”、再次荣获“全国创建文明城市工作先进城市”称号,城市品位不断提升。大力推进新农村建设。加强规划,出台政策,实施帮扶,着力推进农村基础设施和公共服务体系建设,五年累计完成“村村通”道路1043公里,解决11.3万人农村饮水安全,农村远程教育实现“无缝隙”覆盖,农村能源、农业综合开发以及洪庄新农村试点建设走在全省前列。大力推进市矿统筹建设。发挥市矿交错、镇矿伴生优势,推动资源共享、设施共建、产业协调发展,市矿一体化建设进程加快,采煤沉陷区综合治理项目获中国人居环境范例奖。

     和谐淮北加速构建。居民收入全面提高。城镇居民人均可支配收入和农民人均纯收入年均分别增长13.9%和7.6%。城乡居民储蓄存款余额176亿元,年均增长14.3%。民生工程全面展开。实施义务教育经费保障机制改革,建立健全家庭困难学生资助体系,34.7万名学生得到免缴杂费保障;加强薄弱学校建设和农村中小学危房改造,新建和改建校舍18.4万平方米,在全省率先基本消除农村中小学D级危房;普通高考万人本科达线率连续3年居全省前列,职业教育、成人教育、民办教育取得新成绩。积极实施再就业工程,五年新增城镇就业岗位18万个,4.8万名下岗失业人员实现再就业,城镇登记失业率控制在3.8%以内。完善社会保障体系,提高保障标准,城镇职工养老、医疗、失业、工伤、生育保险整体推进;启动实施城镇居民医疗保险,参保居民达24万人。卫生事业健康发展,市公共卫生大厦投入使用,三级公共卫生体系基本形成;建成65家社区卫生服务站,城市医疗服务水平提高;新型农村合作医疗制度覆盖全市,参合率达91.8%;全面实施人均门诊费、出院费和医保目录药品价格三个下降10%,群众看病贵的问题得到缓解。安居工程稳步推进,11万平方米南黎花园经济适用房主体工程完工;1990户居民享受廉租房制度保障。建立和完善城乡低保和农村五保供养制度,9.9万名困难群众得到救助。社会事业全面发展。深化科技体制改革,加强科技服务体系建设,中国(淮北)专利技术展示交易中心挂牌运行。文化事业加快发展,创作生产了一批优秀文艺作品,电视剧《大姐》等获“全国精神文明建设五个一工程”奖;落实网吧管理长效机制,在全国首创市容管理队伍协管网吧新模式;柳孜码头大运河遗址保护和申报文化遗产取得成绩,被列入世界历史文化遗产申报预备名录;开工建设了新图书馆和新广电中心,开通了面向农村的无线微波数字电视系统。体育基础设施建设步伐加快,群众性体育活动日趋活跃,竞技体育水平不断提高。农村计生工作水平稳中有升,城市计生工作在全省继续保持领先。残疾人事业加快发展,2007年被授予“全国残疾人体育先进单位”。产品质量和食品安全、工商管理、贸易促进、双拥、国防、防震减灾等工作继续得到加强。民族宗教、优秀旅游城市创建、机关事务管理、物价、对台、气象、档案、保密、地方志、妇女、儿童、老龄等事业取得新成绩。社会管理全面加强。严格落实安全生产责任制,加大监管力度,开展重点行业和领域专项整治,安全生产形势持续稳定。强化社会治安综合治理,完善矛盾纠纷排查机制,建立应急管理体系,社会安定有序。

     政府建设切实加强。深化行政审批制度改革,取消市级行政许可审批 720项、行政收费225项,全面推行首席代表制和“两集中、两到位”,不仅办事效率大大提高,而且年均减少企业和社会负担6000多万元。加强依法行政,强化规范性文件前置审查,推行行政执法责任制和过错追究制,普法工作连续四年被中宣部、司法部评为先进。自觉接受人大法律监督、工作监督和政协民主监督,密切与社会各界联系,五年共办理人大代表议案10件、建议557件和政协委员提案991件。积极推行政务公开,濉溪县被评为全国政务公开示范点。加强机关效能建设,开通市长热线,提升行风热线,推行限时督察通报等制度,五年累计受理并办结政风类投诉300件,机关作风进一步转变,发展环境进一步优化。全面落实党风廉政建设责任制,加大对商业贿赂和损害群众利益不正风气的治理力度,廉政建设取得新成效。

     刚刚过去的2007年,我们全面贯彻落实科学发展观,解放思想,真抓实干,经济社会发展呈现增长加快、结构优化、效益提高、民生改善的良好态势, 全面超额完成了市十三届人大六次会议确定的各项目标任务。预计地区生产总值258亿元,增长12.8%。财政总收入35.5亿元,增长13.9%。固定资产投资150亿元,增长36%。社会消费品零售总额72.5亿元,增长17%。进出口总额6500万美元,增长21%。城镇居民人均可支配收入11620元,农民人均纯收入3378元,分别增长18.5%和13.8%。

     各位代表,这些成绩的取得,是省委、省政府和市委正确领导的结果,是人大和政协监督、支持的结果,更是全市人民团结奋斗的结果。在此,我谨代表市人民政府,向广大工人、农民、知识分子、机关干部,表示衷心的感谢!向驻淮人民解放军指战员、武警官兵、政法干警,向离退休老同志,向各民主党派、人民团体和社会各界人士,向在我市创业的境内外投资者、建设者,致以崇高的敬意!

     在肯定成绩的同时,我们也清醒地看到存在的差距和不足。一是又好又快发展的基础不够稳固,发展速度在全省居中靠后,传统产业不强,新兴产业发展不快,整体技术含量不高,骨干企业数量不多,经济增长的资源环境成本过高等。二是影响社会和谐的因素仍然不少,社会事业和公共服务投入不足,抗御灾害和社会应急管理能力较弱,居民收入和社会保障总体水平偏低,就业难、上学难、看病难问题仍很突出,控制物价上涨和维护社会稳定也存在较大压力等。三是政府工作还不适应新形势新任务要求,思想解放和改革开放不够深入,影响科学发展、和谐发展的体制机制性障碍还未彻底破除。一些机关重审批处罚、轻监管服务,工作效率不高,执行力不强;一些干部不讲学习、素质较低,作风不实、为政不廉等。对于这些问题,我们将高度重视,采取积极有力措施认真加以解决。

     ****二、乘势而上,奋力加快全面建设小康社会进程****

     未来五年,是我市全面建设小康社会的关键时期,也是落实“十一五”规划、开展“十二五”建设的重要时期。纵观全局,这一时期的发展,机遇与挑战并存。国家建立并实施资源型城市开发补偿机制和资源衰退援助机制;重点支持包括两淮在内的大型煤炭基地建设;中部崛起战略由规划走向启动项目;省委、省政府支持“两淮一蚌”重化工业走廊建设和沿淮城市群培育,都有利于我们加快城市建设、推进经济转型、做大做强煤炭和煤机产业。国企改革、全民创业、招商引资、县域经济发展等积聚的能量加速释放,一批重大项目陆续开工并将投产;广大干部应对复杂局面、解决实际问题的能力和水平不断提高,人民群众谋发展、盼富裕的强烈愿望已化为实际行动,都表明我们跨越发展的主客观条件兼备。与此同时,国家把防止经济增长由偏快转为过热、防止价格由结构性上涨演变为明显通货膨胀作为宏观调控的首要任务,严控贷款规模,严控土地供给,严控新开工项目,提高项目准入门槛,加强节能减排等,使我市以投资拉动发展的约束趋紧;煤电总量实现快速增长需要时日,接续产业、非煤产业形成规模需要一个过程,带动加快发展的大项目少,以及上游产品价格上涨逐步向下游传导、企业效益可能下滑等,也增加了我们加快发展的难度和变数。对此,我们必须抓住机遇,应对挑战,聚精会神搞建设,一心一意谋发展。

     今后五年经济社会发展的指导思想是:认真贯彻党的十七大精神,高举中国特色社会主义伟大旗帜,坚持以邓小平理论和“三个代表”重要思想为指导,全面落实科学发展观,突出科学发展、和谐发展主题,深入实施工业强市、开放带动、创新推动、全民创业、城乡一体化和可持续发展战略,着力优化经济结构、创新发展方式,着力加强社会建设、改善民生,努力建设更具实力、充满活力、富有魅力、文明和谐的现代化工业城市,让全市人民共建美好家园、共享发展成果,为全面建设小康社会打下坚实基础。

     今后五年的奋斗目标和主要工作是:

     (一)紧抓第一要务,实现经济发展实力明显跃升。在优化结构、提高效益、降低能耗、保护环境的基础上,实现地区生产总值、财政收入双翻番,在全省格局中的位次有新提升。大力实施千亿投资计划,确保固定资产投资年均增长30%以上,拉动经济总量扩张和产业层次提升。大力发展现代农业,推进农业产业化经营,实现农业增加值35亿元以上;大力实施工业振兴计划,推进新型工业化发展,实现二产增加值320亿元以上;大力发展服务业,建设现代商贸体系,实现三产增加值170亿元以上,三次产业结构达到 7 :60:33 。做大做强煤电主导产业,加快发展煤化工接续产业,改造提升传统产业,培育发展新兴产业,促进煤电、煤化工和非煤产业“三足鼎立”;开展重点产业带布局和建设,强化配套协作和上下游联动,基本建成煤化工、机械制造、陶瓷建材、纺织服装及高新产业等生产规模超百亿元的产业集群。在重大工业项目布局、基础设施建设、发展特色产业等方面给予支持,促进县区经济加快发展,形成市与县区经济总量相当的新格局。

     (二)坚持改革开放,实现跨越发展动力明显增强。坚定不移地把改革开放引向深入,构建充满活力、富有效率、更加开放、有利于科学发展的体制机制。一是深化国有企业改革,完善事业单位改革,强化投融资体制改革,深入开展全民创业,激活市场主体;完善收入分配制度,规范收入分配秩序,逐步使城乡居民的收入增长与经济增长相适应,增添跨越发展的内生动力。二是进一步拓展对外开放的广度和深度,提高“三区两园”和项目集中区的吸纳力、发展力,发挥淮北矿业集团、皖北煤电等大企业的招商主体作用,强化产业招商,着力引进世界500强企业和国内知名大企业,提高利用外资的层次和水平;培育出口增长源,转变外贸发展方式,深化与国际和沿海发达地区的合作,增添跨越发展的外源动力。三是转变政府职能,理顺和规范部门分工,提高行政效能,更好地为市场主体和人民群众服务;深化行政审批制度改革,规范市场经济秩序,健全诚信体系,优化发展环境。

     (三)创新发展方式,实现持续发展能力明显提升。加强自主创新和生态环境建设等,形成有利于可持续发展的经济发展方式和社会生活方式。实施创新推动战略,促进经济发展由主要依靠增加物质能源消耗向主要依靠科技进步、劳动者素质提高和管理创新转变;发挥专利技术交易中心作用,实施产学研结合,建立原始创新、集成创新和引进消化吸收再创新机制,培育更多具有较强竞争力的创新型企业,大幅度提高科技对经济增长的贡献率。实施人才兴市战略,引进、培育和用好人才,并支持淮北煤炭师范学院创建淮北师范大学。强化节水型社会和节水型城市建设,强化土地节约集约利用,强化电源点、电网建设和用电体制机制改革,提升要素支持发展的能力。把建设资源节约型、环境友好型社会放在工业化、现代化发展战略的突出位置,完善政策机制,落实目标责任,合理开发、节约使用和综合利用各种资源,推动全社会、各园区和企业大力发展循环经济,强力推进节能减排,切实加强生态建设,实现青山常在、绿水长流、永续发展。

     (四)统筹城乡建设,实现协调发展水平明显提高。大力推进城市化和城市现代化,形成城乡一体、共同繁荣的新格局。落实国务院批准的城市建设总体规划,大力推进老城区改造,高标准建设新城区;加强山水环境建设和文明城市创建,形成“两山环绕、六湖珠连、城在山中、水在城中”的山水城市格局,建设“双百双宜”城市,争创全国文明城市、全国绿化模范城市。打破城乡壁垒,促进各类要素双向流动,推进农村基础设施布局与城市对接、城市公共服务向农村延伸,抓好“乡镇文化站、农村电影放映、文化信息资源共享、广播电视村村通、农家书屋”五大工程,加快新农村建设和城镇化进程。完善发展规划,建立联动机制,提高市矿一体化建设的协调性和推进度,共建共享现代文明。

     (五)促进社会和谐,实现人民生活质量明显改善。坚持以人为本,切实加强以改善民生为重点的社会建设,努力使人民群众学有所教、劳有所得、病有所医、老有所养、住有所居,富裕程度普遍提升,生活质量明显改善,实现城镇居民人均可支配收入和农民人均纯收入年均分别增长12%以上和10%,覆盖城乡的公共服务体系和社会保障体系基本建成。把政府新增财力更多地用于民生,推进以解困和公平为主向全面提升人民生活品质转变。促进大文化的发展与繁荣,加强文化基础设施建设,完善多层次、多体制的文化网络,发展文化产业,丰富人民精神文化生活。建立健全人口和计划生育工作协同推进机制,继续稳定低生育水平,实现人口与资源、环境、经济发展相协调。加强社会管理,维护安全稳定,进一步增强人民群众的安全感、幸福感和满意度。

    ****三、积极开局,切实做好2008年的各项工作****

     2008年是新一届政府的开局之年,也是我市实施“十一五”规划的关键之年。今年工作的总体要求是:认真贯彻党的十七大、中央经济工作会议和市委全会精神,全面落实科学发展观,着力优化经济结构,积极推进改革开放,更加注重改善民生,切实加强生态建设,推动经济社会又好又快发展。

     经济社会发展主要预期目标是:地区生产总值增长13%以上,财政收入增长13%以上,社会消费品零售总额增长15%以上,固定资产投资增长30%以上,规模以上工业增加值增长16%,城镇居民人均可支配收入增长10%,农民人均纯收入增长10%左右,城镇登记失业率控制在4%以内,单位生产总值能耗下降4.4%,主要污染物排放量减少2%,人口增长完成省下达指标。

     为实现上述目标,我们将重点抓好九个方面的工作:

     (一)大力推进工业强市,加快新型工业化进程

     深入实施“3588”工业振兴计划,全力推进工业项目攻坚年活动,着力优化工业经济结构,提高工业经济增长质量。

     做大工业总量。突出抓好主导产业和新兴产业发展。加快改造和新建一批高产高效现代化矿井,提高采掘机械化程度,稳步扩大煤炭生产,实现新增产能300万吨。加快推进临涣煤焦化项目建设和生产能力形成,实现主营销售收入30亿元。发展煤矿机械制造业,实现主营销售收入20亿元。推进华孚色纺、维科印染等企业技术改造、产品更新和产能提升,支持民营纺织发展,实现产值增长20%以上。发展壮大陶瓷建材工业,新增建材企业20家,实现产值增长40%。发展农副产品加工业,做大做强饮料酒、畜禽加工、粮食加工等产业,实现增加值增长20%。

     培育发展后劲。建立市领导挂帅、有关部门跟踪服务的重大项目推进机制,加快项目建设。一是抓好在建项目建设,确保孙疃、五沟矿井和宝迪食品等项目竣工投产,杨柳、刘店矿井实现联合试运转,做好青东矿井等项目征地、核准以及开工建设工作。建成临涣焦化一期,启动建设焦化二期,确保临涣煤泥矸石电厂2×30万千瓦机组并网发电。抓好丰盛泰重工等非煤产业重点项目建设。二是尽快开工建设成熟项目,加快建设淮矿集团焦油加工、粗苯加氢精制等项目,启动电源点建设,争取国安电厂2×60万千瓦机组尽早开工。三是加强项目库建设,策划论证并集中吸纳一批成长性高的项目入库管理,力争15个重大项目列入国家计划,15个项目列入省技术改造“百千工程”,35个项目列入省技术创新专项计划。

     提高运行效益。扶优扶强,积极支持淮北矿业集团、皖北煤电、国安电力等省属企业加快发展,认真服务好口子酒业、维科印染、华孚色纺等骨干企业发展,努力扩大煤炭、电力、纺织、酿酒等主导产业的生产能力,推动规模化、集群化发展。培育主体,确保新增规模以上企业150家,总数突破400户。扭亏增盈,重点引导劣势企业加强管理、改善运营。

     (二)大力推进农民增收,建设社会主义新农村

     坚持统筹城乡发展,以增加农民收入为核心,加大支农惠农政策落实力度,大力推进农村经济增长、社会进步和环境改善。

     加快推进新农村建设。落实新农村建设规划和产业发展规划,与采煤塌陷村庄搬迁相结合,突出抓好“五镇十村”试点建设。加快农村基础设施建设,新建改建农村公路200公里,建成 3万亩标准农田,完成3万户广播电视村村通工程,解决10万农民饮水安全。推进教育、文化、医疗等公益事业向农村延伸,促进公共服务均等化。加强民主政治建设,完善乡村治理机制。

     着力增强农业综合生产能力。推进农业标准化生产、农产品质量安全和技术推广服务体系建设,提高为农服务水平。继续实施小麦高产攻关活动,切实加快高效经济作物种植区建设,大力发展规模养殖业。以宝迪、六和、曦强等企业为龙头,发展壮大畜产品加工业;以天宏、鲁王等企业为龙头,推进粮食加工业向精深加工转变。发展专业合作组织,提高农民组织化程度。强化农民技能培训,发展劳务经济,确保劳务输出新增1万人。

     (三)大力推进三产发展,提高服务业发展水平

     认真落实扩大消费需求方针,繁荣城乡市场,协调拉动经济发展和民生改善。

     加强市场体系建设。进一步理顺商贸管理体制,修编和实施城市商业网点规划。着力提升以淮海路为中心的老城区和以商业步行街为中心的新城区的商业圈功能,构建城市商业中心新格局。继续实施社区商业“双进”工程,形成具有购物、餐饮、生活服务等多功能的社区商业体系。继续实施“万村千乡”和“双百市场”工程,推动农村商业网点建设。走市场带动产业发展之路,重点建好矿业设备博览城等市场和宝迪等物流配送中心,培育好新惠康、中瑞商贸、金信农资等重点流通龙头企业。

     大力发展服务业。着眼服务业和加工制造业的相互融合,加快培育和完善资本、土地、劳动力、技术等生产要素市场,大力发展信息咨询、技术交易、金融保险、仓储物流等生产服务业。顺应消费结构升级,加快发展商贸流通、餐饮等传统服务业和文化娱乐、教育培训、体育健身等新型生活服务业,建成口子国际大酒店等一批较高星级酒店,建设一批经济型酒店,加快开发区、园区的生活服务设施建设。推进华联、苏果等向更大范围拓展,争取国际知名连锁企业集团落户我市,确立连锁经营方式的主导地位。加强旅游业基础设施建设,发展红色旅游、生态旅游,打造精品景区和线路,推进跨区域合作,培育旅游产业。

     (四)大力推进城市建设,增强城市综合竞争力

     以创建全国文明城市为载体,以提升人居环境和支撑经济发展能力为目标,充分发挥规划的龙头作用,加大工作力度,强力推进城市化和城市现代化。

     科学制定并严格执行规划。以城市总体规划为统领,完善老城区规划和新城区规划,编制完成南湖景区、合徐高速公路出入口区、人民路东段等重点地区规划,做好孟山路景观、淮海商场、淮北火车站等城市重要节点处的修建性详细规划,积极开展铁路两侧改造规划编制,并严格执行。

     强力推进城市基础设施建设。围绕扩大规模、增强功能、提升品位、彰显特色,加强重大基础设施项目建设,提高城市的承载力、吸纳力,改善人居环境。坚持老城区改造和新城区建设并重,按照降低密度、完善配套、优化环境的要求,重点推进“城乡结合部”和“城中村”改造,优化老城区功能;切实加快新区建设步伐,提升综合承载力和服务功能。一是启动实施城市道路新建工程,建设沱河东路、栗苑路等道路,推进相濉、相烈、相杜一体化;扩建西外环路,改建省道101五宋段,解决过境交通与城市交通相互干扰的矛盾。二是启动实施新城区截污引水环境综合治理工程,建设西流河引南湖水工程,改善新城区水质量;对相山南路、长山路沟排水系统进行改造,彻底消除新城区积水隐患。三是启动建设城市风景区和景观工程,重点抓好相山公园、国家矿山公园等6个公园的改造和建设,启动化家湖、南湖、龙脊山景区和世纪广场、人民路等景观建设,彰显山水园林城市特色。四是加强城市公用事业建设,新建公交场、站和中转枢纽13个,新辟线路19条;实施天然气工程,全面改造老城区供气管网,置换天然气用户8万户,发展新用户12万户。

     提高城市管理水平。深入开展群众性精神文明创建活动,切实加强市民教育。创新城市管理机制,加强数字化城市管理,依法整治环境。加快环卫基础设施配套建设,力争建成医疗废弃物处置中心并投入使用,启动建设垃圾发电厂。突出规范管理、热情服务,推进文明单位建设。突出城乡联动、矿镇共建,推进文明村镇建设。突出安全、温馨、文化,推进文明社区建设。

     (五)大力推进各项改革,创新发展的体制机制

     深化重点领域和关键环节改革。深化国企改革,对改制企业跟踪服务、加强监管,帮助建立、完善现代企业制度和新型劳资关系,引导企业开展招商引资和技术改造;推进大型企业主业整合、主辅分离和战略重组,促进雷鸣科化、恒源煤电等增资扩股,支持淮北矿业集团煤业、中煤矿建加快上市,培育口子酒业等地方优势企业上市。深化财政改革,依法加强税收和非税收入征管,扩大财政收入总量;调整优化支出结构,重点支持民生、城市、新农村和社会事业建设;积极推进预算制度改革,建立编制、执行、监督管理新模式;逐步取消预算单位收入过渡账户,将部门所有国有资产经营收入统一纳入财政管理。深化金融体制改革,构建政银企合作协调机制和金融机构支持地方经济建设考核激励机制;加强金融产品创新,积极引进非国有金融机构来淮设立分支机构;优化金融生态环境,防范化解金融风险。深化投融资体制改革,整合政府融资平台,建立决策、执行、监督分离和“借用还”良性运行机制。强力推进事业单位改革,基本实现经营性事业单位退事转企,实行全员聘任制。深化农村综合改革,探索农村土地集约经营改革。

     深入开展全民创业。推进公平准入,破除体制障碍,清理和压减收费项目,规范各类检查,优化发展环境;实施中小企业成长工程,办好2008专利技术推介会,促进民营经济发展,新增个体工商户3800户、私营企业650家、从业人员15000人。

     规范市场经济秩序。加强价格监测和市场监管,及时完善和落实因基本生活必需品价格上涨对低收入群众的补助办法;进一步运用价格调节基金,促进副食品基地建设,稳定粮、油、肉等主要副食品价格。进一步加大食品药品、交通运输、农业生产资料、建筑等市场秩序整顿力度,严厉打击传销、变相传销和制假、售假等行为,维护公平竞争的市场秩序。继续推进企业信用分类监管,加大对违法违规企业的惩戒力度。

     (六)大力推进对外开放,提升经济发展外源动力

     提高招商引资的质量和水平。围绕实现利用内资150亿元、外资1亿美元目标,广泛开展大招商、招大商活动,扩大招商总量,优化利用外资结构,推动产业升级。围绕我市重点推介的煤化工、煤矿机械制造、纺织服装、陶瓷建材、农产品深加工等产业项目,瞄准产业转移的重点领域和区域,紧紧盯住世界500强和国内外知名企业,强力开展重点招商。创新招商方式,突出产业招商,配套实施企业招商、专业招商、驻点招商和中介招商,举办好2008煤矿机械投资贸易洽谈会,组织好国内外招商推介活动,提高招商实效。进一步落实招商项目调度例会制度,完善招商引资奖励和考核机制、市级领导联系重点招商项目机制、招商引资精细化管理机制和引进项目协调联动机制。坚持引资和选资并重,把好投资强度与环保两个关口,加强产业项目基础性工作,抓好策划、包装、推介和跟踪服务。

     强力推进园区的建设与发展。重点推进市开发区和县区园区加大招商引资力度,完善配套服务功能,开展体制机制创新,实施封闭式运行,力争招商引资总量翻一番。加强县区开发区和园区基础设施建设,把住产业特色、投资强度、环境保护等关口,推动五大重点非煤产业向园区集聚,实现错位发展。

     努力扩大对外贸易。筹建淮北出入境检验检疫局,推动外贸出口便捷化。用足用活国家和省出口退税、中小企业开拓国际市场资金申报等政策,促进加工贸易转型升级。立足以质取胜,调整出口结构,重点加快培育以维科印染、飞亚纺织等为龙头的纺织服装出口基地,加速形成以福瑞祺为龙头的农产品出口基地,力争进出口总额突破1亿美元。

     (七)大力推进区域统筹,支持县区经济加快发展

     县区经济不强,制约全市经济跨越发展。我们将采取综合措施,促进县区加快发展,着力培育新的经济增长极。完善县区发展推动机制。制订鼓励县区发展的政策措施,调动加快发展的积极性。完善县区发展分类考核办法,强化各自发展责任。建立县区发展联席会制度,协调解决重大问题。

     赋予县区更多发展自主权。按照事权合理、责权统一、重心下移、有利发展的原则,进一步扩大县区经济社会管理权限。凡省下放到县区的权限坚决下放,凡市里能下放的权限一律下放。同时,适时适度调整市、县(区)相关利益格局。

     加大对县区扶持力度。实施市重大工业项目向县区倾斜布局,对县区优势产业和企业给予支持,用全市工业经济发展专项资金对县区工业园区和集中区重点项目进行奖励。加大转移支付力度,实施社会保障全市统筹,对县区实施民生工程拨付更多的资金,推进公共服务均等化。帮助县区加强基础设施建设,多元融资支持每个县区8000万元左右建设城镇和工业园区。同时,积极帮助协调各类商业银行扩大对县区的信贷投放。

     (八)大力推进可持续发展,促进资源节约环境友好

     启动国家级循环经济试点市建设。研究省主体功能区规划编制动态,加快编制循环经济实施方案,建立和完善推进机制,支持淮矿集团申报“国家循环经济试点企业”。把调整经济结构作为资源循环利用的重中之重,支持和引导企业进行技术改造,建立健全资源节约管理制度,形成循环型的生产模式,重点加快推进淮矿集团临涣工业园区上下游产业和废弃物综合利用、国安电力能量系统优化、维科印染余热回收利用、城市污水处理中水回用等一批资源节约和环境保护项目建设。

     强力推进节能减排。建立健全责任体系,尽快形成以政府为主导、企业为主体、全社会共同推进的工作格局。综合运用财税、价格、信贷等政策,严格新上项目的节能评估和环境评审,严格核发排污许可证,有效遏制高耗能、高排放行业过快增长,坚决淘汰落后生产能力。强化责任落实,认真做好节能减排统计、监测和考核工作,编制实施行业和企业节能减排规划,建立节能减排和环境公告制度。强化企业社会责任,突出抓好煤炭、电力、建材等重点行业、重点领域的节能减排工作。

     加强资源环境建设。加快城市固体废弃物无害化处理,强化生产生活噪声监管,治理农业面源污染,积极创建国家环保模范城市。大力推进土地开发复垦整理,建立包括“空心村”和“关闭企业”在内的闲置和低效利用土地退出机制,提高土地集约利用水平。积极推进节水型社会和节水型城市建设,加快 “淮水北调”工程和临涣工业园引水工程进度。认真搞好荒山绿化、林带林网建设和塌陷区生态恢复,创建全国绿化模范城市。

     (九)大力推进社会建设,积极构建和谐淮北

     持续实施民生工程。通过积极向上级争取,调整财政支出结构,筹集5亿元用于民生工程建设。

     继续实施均衡教育工程。完善义务教育经费保障机制,保证教师待遇、学校运转和校舍维修改造资金需求,妥善解决原企业移交学校遗留问题。重点完成梅苑等学校建设,启动淮北实验高级中学异地建设,解决主城区教育资源短缺,尤其是新城区上学难的问题。建立和完善农村中小学校舍维修维护长效机制,逐步缩小城乡、校际之间的差距。建立中小学教师补充长效机制,提升教学质量。建立和完善家庭经济困难学生资助体系,保障贫困家庭学生有平等接受教育的权利。

     继续实施扩大就业工程。落实省统筹城乡就业试点市工作,坚持发展经济与促进就业互动,健全覆盖城乡的就业服务体系,规范和协调劳动关系,投资853万元建设市级创业基地,促进以创业带就业,构建大就业格局,确保新增城镇就业3万人,下岗失业人员再就业7500人。

     继续实施社会保障工程。完善城镇企业职工保险制度和市级统筹配套政策,落实城镇集体企业未参保退休人员基本生活保障办法,做好企业退休人员养老金标准调整和发放工作。大力推进养老保险扩面工作,确保养老保险参保人数达到18万人。加强失业、工伤、生育等保险工作,扩大参保覆盖面。实施被征地农民养老保险制度,提高参保水平。完善城乡低保制度,在全覆盖的基础上逐步提高低保标准。

     继续实施健康卫生工程。深化医疗保险制度改革,确保城镇职工医疗保险参保人数达到36万人,城镇居民医保参保人数达到30万人,基本实现全覆盖。继续做好新型农村合作医疗全覆盖后的后续工作,完善政策、提高质量、安全运作。加强卫生服务体系建设,开工建设市人民医院病房大楼,支持建设25个社区卫生服务站、9所乡镇卫生院和100所村卫生室。

     继续实施居民安居工程。强化政府在稳定房价和住房保障方面的责任,提高中小套型普通商品房和经济适用房在住房供应中的比例,加大房地产市场规范和监管力度。把解决中低收入家庭住房困难与城市拆迁安置工作统一起来,加快经济适用房建设,5月底交付使用南黎花园,年底前完成西山经济适用房一期楼栋主体封顶。健全廉租住房制度,实行动态管理,实现应保尽保。

     继续实施贫困救助工程。不断完善城乡特困群众医疗救助制度,适度提高农村五保对象供养标准。同时,加强敬老院和老年服务机构建设,努力提高集中供养率和社会化服务水平。

     切实维护社会稳定。大力推进平安淮北建设,加强基层基础工作,构建社会治安综合防控体系,严厉打击刑事和经济犯罪。加强和改进信访工作,畅通利益诉求渠道,不断完善社会矛盾纠纷排查调处机制,坚决纠正土地征用、城镇拆迁、企业改制中侵害群众合法权益的行为。建立健全安全生产监管体制机制,强化企业主体责任,突出抓好矿山、消防、交通等重点领域的安全监管,杜绝重特大事故发生。建立和完善各级地企联动的应急管理体系,提高突发事件处置能力。

     全面发展社会事业。突出自主创新、普及转化两个重点,创办产业研发中心和孵化基地,并与20所院校和科研院所建立合作伙伴关系。加快新广电中心和新图书馆建设,投入使用新体育场,做好优秀作品创作和柳孜码头大运河遗产申报工作;推动广播电视、文艺团体等进行市场化改革,开展产业化经营。大力发展残疾人事业,认真抓好外事、侨务和对台事务工作,做好人才、档案、保密、地方志、气象、地震、电讯、新闻出版、民族宗教、机关事务管理、住房公积金管理等工作。发展老龄事业,实施妇女儿童发展纲要,维护妇女权益,加强未成年人保护。深入开展“双拥”活动,加强国防动员和后备力量建设,巩固军政军民团结。

     ****四、执政为民,强力推进政府管理创新****

     新形势、新任务,对政府工作提出了新的更高的要求。我们将深入开展“创新促发展、执行看落实”解放思想主题实践活动,加快职能转变,推进管理创新,提高行政效能,努力为人民服务,对人民负责,受人民监督,让人民满意。

     (一)规范行政行为,建设法治政府。坚持“市场出效率、政府保公平”原则,以促进社会公平、正义、和谐和维护群众利益为重点,切实加强政府法制建设。认真落实行政执法责任制和过错责任追究制,加强执法检查,切实解决执法随意性较大、自由裁量权过大和造成过错不赔偿的问题,保证公正执法、文明执法。认真接受市人大及其常委会的法律监督和工作监督、市政协的民主监督,听取社会各界的意见和建议,落实人大决议,办理人大代表议案、建议和政协委员提案。认真实施政务信息公开条例,全面推行政务公开和公共企事业单位办事公开,健全公示、听证、新闻发言人等制度,切实解决一些权力部门看得见管不着,一些行业协会和中介机构依附权力乱收费的问题,提高政府工作透明度,保障广大群众的知情权、参与权和监督权。

     (二)坚持立言力行,建设诚信政府。树立正确的政绩观,说实话、办实事、求实效,防止弄虚作假、虚报浮夸,杜绝“形象工程”和“政绩工程”,使我们的各项工作经得起实践、群众和历史的检验。加快建立健全科学的政务决策和公信体系,坚持一切从实际出发,办不成的事情不说,脱离实际的目标不定,切实做到言必行、行必果。凡是政府已经出台的政策和承诺的事情,执行中确保不打折扣,坚决杜绝对外来投资者承诺时振振有词、执行时吃拿卡要的现象。充分发挥政府诚信的导向性作用,深入开展争创“优秀诚信机关”活动,实现诚信执政、诚信服务,并以政府诚信来推动和规范商务诚信、社会诚信。

     (三)强化服务职能,建设责任政府。围绕促进科学发展、和谐发展,进一步优化公共资源配置,加强公共设施建设,完善社会管理制度,提高社会管理水平,着力解决人民群众最关心、最直接、最现实的利益问题。继续推进以作风和行风建设为主要内容的政风建设,坚持行政问责与行政监察、审计监督相结合,努力破除一些部门、单位缺乏大局和责任意识,为了自身和小团体利益,不惜损害公众权益和投资者利益的积弊,坚决治理“中梗阻”,严肃查处破坏和干扰发展环境的行为。建立层级清晰的责任体系,努力改变一些部门和单位有利就争、麻烦就推,有事没人做、有人没事做,致使许多工作有部署无落实、有责任无追究的状况,做到对组织、对人民负责,让社会满意。

     (四)优化管理流程,建设效能政府。坚持效率优先、讲求务实,保证政府工作的高效有序运转。强化全局意识,努力消除有人出题、无人作文的弊端,坚决杜绝推诿扯皮、各行其是的现象,不断完善重大事项和重点工作落实机制,确保政令畅通。全面推行岗位责任制,逐步理顺部门职能分工,优化工作流程,精简会议和文件,大力推进电子政务建设,不断提高行政效率。进一步减少和规范行政审批事项,继续加强行政服务中心建设,全面推行“两集中、两到位”、首席代表制和并联审批制,提高窗口办结率。坚持把群众公认作为政府绩效评估的根本原则,加快建立科学合理的绩效评估办法和指标体系,硬化绩效评估的导向作用和激励约束作用,高标准、高效率地做好每一件事情,不断提高政府的执行力。

     (五)预防惩治并重,建设廉洁政府。坚持从严治政,认真贯彻标本兼治、综合治理、惩防并举、注重预防的方针,全面落实廉政建设责任制。从政府班子做起,带头反对铺张浪费和大手大脚,带头抵制拜金主义和奢糜之风,严禁做事不计成本、不惜代价,一味贪大求快、急功近利、奢侈浪费。深入开展商业贿赂治理,重点整治工程建设、土地出让、产权交易、医药购销、政府采购、资源开发和经销领域的违法违纪行为,坚决纠正损害群众利益的不正之风。加强廉政建设和反腐败工作,强化对公务员队伍的警示教育和监督管理,防止公务员道德失范和腐败现象发生。

     (六)注重提升能力,建设学习政府。教育全体政府公务人员特别是领导干部切实增强学习意识,改变整天忙于事务和应酬,不研究工作、安于现状的状况,树立在学习中工作、在工作中学习的新理念。通过在职自学、“菜单式”选学、网络培训等多种方式,鼓励个体学习,倡导组织学习,构建学习型政府的平台。加强调查研究,深入基层、深入群众,虚心向群众学习、向实践学习,了解和把握真实情况,在具体实践中探索和完善发展思路,在破解难题中增长知识和才干。把学习培训与公务员的评先评优、晋职晋级结合起来,把公务员参加学习培训情况作为实绩考核的重要内容,建立学习型政府的长效机制。弘扬理论联系实际、学以致用的良好学风,着眼于现实问题的解决和未来事业的筹谋,努力提高抓关键、破瓶颈的水平,切实增强驾驭市场经济、处理复杂问题和引领科学发展、和谐发展的能力。

     各位代表!肩负时代的使命和人民的重托,我们深感事业光荣、责任重大。让我们在市委的坚强领导下,在市人大、市政协和社会各界的监督支持下,以党的十七大精神为指导,以科学发展观为统领,与时俱进,真抓实干,为加快建设“更具实力、充满活力、富有魅力、文明和谐”的现代化工业城市而努力奋斗!
